# Changes in the Intestinal Histomorphometry, the Expression of Intestinal Tight Junction Proteins, and the Bone Structure and Liver of Pre-Laying Hens Following Oral Administration of Fumonisins for 21 Days

**DOI:** 10.3390/toxins13060375

**Published:** 2021-05-25

**Authors:** Ewa Tomaszewska, Halyna Rudyk, Piotr Dobrowolski, Janine Donaldson, Izabela Świetlicka, Iwona Puzio, Daniel Kamiński, Dariusz Wiącek, Volodymyr Kushnir, Oksana Brezvyn, Viktor Muzyka, Renata Doraczyńska, Siemowit Muszyński, Ihor Kotsyumbas

**Affiliations:** 1 Department of Animal Physiology, Faculty of Veterinary Medicine, University of Life Sciences in Lublin, Akademicka St. 12, 20-950 Lublin, Poland; iwona.puzio@up.lublin.pl; 2 State Scientific Research Control Institute of Veterinary Medicinal Products and Feed Additives, Donetska St. 11, 79000 Lviv, Ukraine; galusik.77@gmail.com (H.R.); wolodjak@gmail.com (V.K.); brezvun@gmail.com (O.B.); muzyka@scivp.lviv.ua (V.M.); dir@scivp.lviv.ua (I.K.); 3 Department of Functional Anatomy and Cytobiology, Faculty of Biology and Biotechnology, Maria Curie-Sklodowska University, 19 Akademicka St., 20-033 Lublin, Poland; piotr.dobrowolski@umcs.lublin.pl; 4 School of Physiology, Faculty of Health Sciences, University of the Witwatersrand, 7 York Road, Parktown, Johannesburg 2193, South Africa; janine.donaldson@wits.ac.za; 5 Department of Biophysics, Faculty of Environmental Biology, University of Life Sciences in Lublin, Akademicka St. 13, 20-950 Lublin, Poland; izabela.swietlicka@up.lublin.pl (I.Ś.); renatadoracz@gmail.com (R.D.); 6 Department of Crystallography, Faculty of Chemistry, Maria Curie-Sklodowska University, Maria Curie-Skłodowska Sq. 2, 20-031 Lublin, Poland; daniel.kaminski@umcs.lublin.pl; 7 Department of Physical Properties of Plant Materials, Bohdan Dobrzański Institute of Agrophysics of the Polish Academy of Sciences, Doświadczalna St. 4, 20-290 Lublin, Poland; d.wiacek@ipan.lublin.pl

**Keywords:** fumonisins, pre-laying hen, gut, liver, bone

## Abstract

Fumonisins (FB) are metabolites found in cereal grains (including maize), crop products, and pelleted feed. There is a dearth of information concerning the effects of FB intoxication on the intestinal histomorphometry, the expression of intestinal tight junction proteins, and the bone structure and liver in pre-laying hens. The current experiment was carried out on hens from the 11th to the 14th week of age. The hens were orally administered an extract containing fumonisin B1 (FB1) and fumonisin B2 (FB2) at doses of 0.0 mg/kg b.w. (body weight), 1.0 mg/kg b.w., 4.0 mg/kg b.w., and 10.9 mg/kg b.w. for 21 days. Following FB intoxication, the epithelial integrity of the duodenum and jejunum was disrupted, and dose-dependent degenerative changes were observed in liver. An increased content of immature collagen was observed in the bone tissue of FB-intoxicated birds, indicating intensified bone turnover. A similar effect was observed with regards to the articular cartilage, where enhanced fibrillogenesis was observed mainly in the group of birds that received the FB extract at a dose of 10.9 mg/kg b.w. In conclusion, FB intoxication resulted in negative structural changes in the bone tissue of the hens, which could result in worsened bone mechanics and an increase in the risk of bone fractures. Fumonisin administration, even at a dose of 1.0 mg/kg b.w., can lead to degradation of the intestinal barrier and predispose hens to intestinal disturbances later in life.

## 1. Introduction

Large-scale animal nutrition differs significantly from the way animals feed in the wild. Nutrition is an important factor in the maintenance of animal health, and errors in the preparation and storage of feed can result in significant economic losses. Cereal meal, commonly used in animal nutrition, regardless of the form of processing, especially in favorable climatic conditions, may be infested with fungi. Improper storage of contaminated microbiological grain promotes the further development of fungi, which, in turn, produce toxic compounds. Maize serves as a good substrate for the growth of fungi. *Aspergillus*, *Fusarium*, and *Penicillium* produce toxins such as ochratoxin, trichothecenes, aflatoxin, zeralenon, and fumonisin. Fumonisins (FB) are heat-resistant metabolites found in cereal grains (including maize) and crop products including pelleted feed. The most important FB are B1 and B2, which can be found in over 50% of investigated feed and feed raw material samples [1,2]. The toxicity of fumonisins, specifically that of B1, is related to disrupted sphingolipid synthesis [3]. The presence of fumonisins B1 and B2 in animal feed is regulated by EU legislation, which allow for the presence of 20 mg/kg feed for poultry [4]. A toxic dose of FB, as well as the various clinical implications, differ between animal species and are also dependent on the route of FB administration as well as the sex and age of the animals [5]. Horses, pigs, sheep, and rodents are more sensitive to FB compared to other animals and display non-species-specific symptoms, including hepatic or kidney toxicity [5,6,7,8], as well as some species-specific symptoms on target organs (e.g., the brain in horses and the lungs in swine) [9]. The bioavailability and toxicity of FBs in ruminants and poultry is poor compared with other species. Birds are quite resistant towards the deleterious effects of FBs. In poultry, morphological and functional changes following FB toxicity are also dependent on the avian species. Turkeys and ducks are more susceptible to FB toxicity than broiler chickens, in which doses of up to 300 mg/kg feed can induce clinical toxicity [9].

Because avian species vary in their sensitivity to FB and because there is very limited knowledge of the effects of FB in laying hens, the current study was designed to evaluate the structural changes in the gut–bone axis of hens that were administered FB for 21 days during the pre-laying period. The parameters analyzed included the content of bone microelements, damage to articular cartilage, the assessment of collagen structure (distribution) in bone and gut tissues, basal histomorphometrical analysis of gut and trabecular bone, and the expression of gut barrier proteins. The current study enabled us to assess the structural changes in the intestinal tract, the primary site of exposure to FB, the proper physiological function of which is linked not only to the general homeostasis of the body but also to that of the bones, which function (the medullary bone) to store calcium phosphate and other minerals for eggshell formation.

## 2. Results

**Figure 1:**
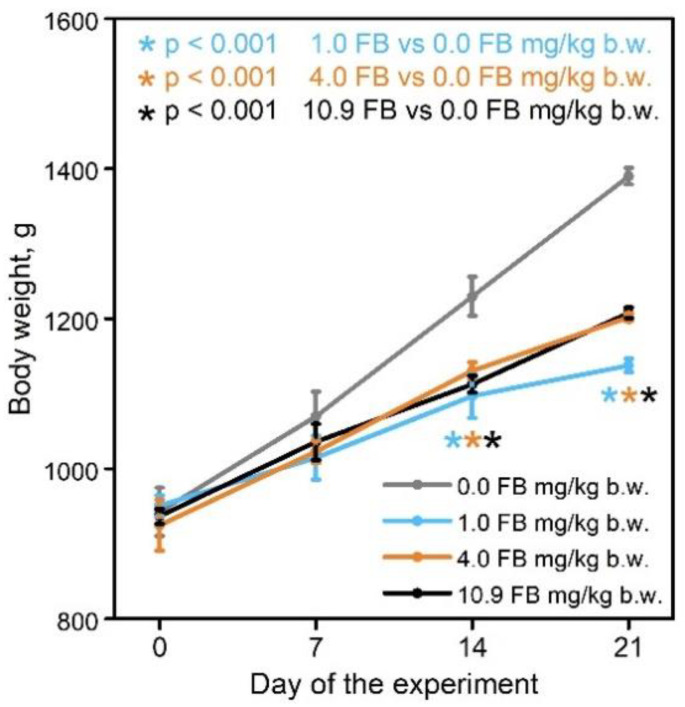
The effect of fumonisin intoxication on body weight gains of pre-laying hens. The data are expressed as mean ± standard error (SE). Significance was established for fumonisin-intoxicated groups versus the control group (no FB) using a one-way ANOVA followed by a Dunnett’s post-hoc test (normally distributed data) or a Kruskal–Wallis ANOVA with a Dunn’s post-hoc test (for pairwise comparisons with at least one non-normally distributed dataset).

There were no cases of mortality following administration of 1.0, 4.0, or 10.9 mg FB/kg b.w. for 21 days. At the end of the experimental period, hens in the control group had the highest final body weight (1390 ± 31 g), whereas the final body weights of hens in all FB-intoxicated groups were significantly lower (Figure 1; *p* < 0.001 for all groups) compared to the control hens, irrespective of FB dose (1138 ± 25 g, 1202 ± 15 g, and 1208 ± 21 g for the 1.0, 4.0, and 10.9 FB groups, respectively).

### 2.1. Basal Blood Morphology and Blood Serum Biochemical Analysis

**Figure 2:**
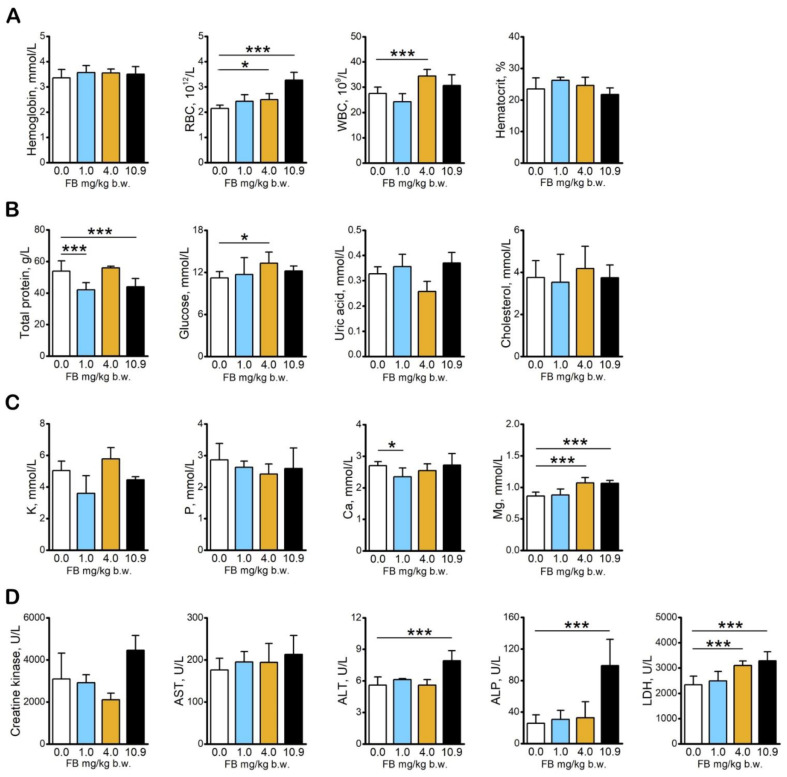
The effect of fumonisin intoxication on basal blood morphology and blood serum biochemical analysis in pre-laying hens. (**A**) Basal morphology analysis; (**B**) blood serum concentration of total protein, glucose, uric acid, and cholesterol; (**C**) blood serum concentration of selected elements (K, P, Ca, Mg); (**D**) activity of creatine kinase, aspartate transaminase (AST), alanine aminotransferase (ALT), alkaline phosphatase (ALP), and lactate dehydrogenase (LDH). Data are expressed as mean ± standard error (*n* = 8 in each group). Significance was established for fumonisin-intoxicated groups versus the control group (no FB) using a one-way ANOVA followed by a Dunnett’s post-hoc test (normally distributed data) or a Kruskal–Wallis ANOVA with a Dunn’s post-hoc test (for pairwise comparisons with at least one non-normally distributed dataset); * *p* < 0.05; *** *p* < 0.001.

Basal blood morphological analysis revealed an increase in the number of erythrocytes in the hens administered with 4.0 mg/kg b.w. and 10.9 mg/kg b.w. FB as well as in the number of leukocytes in the 4.0 mg/kg b.w. FB-intoxicated group (Figure 2A) compared to that observed in the control hens. The total protein concentration was significantly decreased in hens in the 1.0 mg/kg b.w. and 10.9 mg/kg b.w. FB-intoxicated groups compared to the control hens. Glucose concentrations were significantly increased in hens in the 4.0 mg/kg b.w. FB group compared to that of hens in the control group (Figure 2B). Intoxication with 4.0 mg/kg b.w. and 10.9 mg/kg b.w. FB doses significantly increased Mg concentrations, and intoxication with 1.0 mg/kg b.w. of FB significantly decreased calcium concentrations compared to those observed in the control group (Figure 1C). Alkaline phosphatase (ALP) and alanine aminotransferase (ALT) activity was significantly increased in growing hens following administration of 10.9 mg/kg b.w. of FB, whereas lactate dehydrogenase (LDH) activity was increased following FB intoxication at a dose of 4.0 and 10.9 mg/kg b.w. compared to the control hens (Figure 2D). No other changes in serum biochemical parameters were observed. Almost all biochemical parameters were in range of physiological levels except ALP and LDH, especially in the group intoxicated with FB at the dose of 10.9 mg/kg b.w [10,11].

### 2.2. Results of Intestine and Liver Analyses

**Figure 3:**
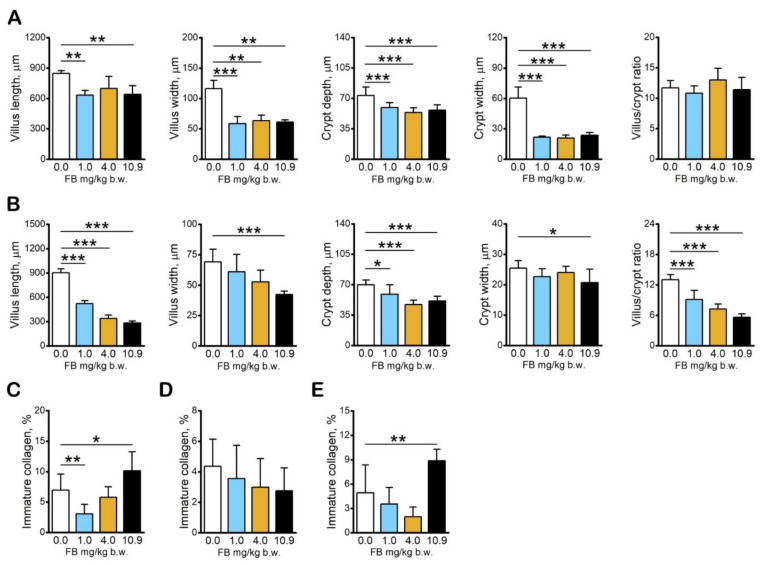
The effect of fumonisin intoxication on intestine histomorphometry and immature collagen content in intestine and liver tissue in pre-laying hens. (**A**) Histomorphometric analysis of duodenum mucosa; (**B**) histomorphometric analysis of jejunum mucosa; content of thin, immature collagen fibers in sections of (**C**) duodenum, (**D**) jejunum, and (**E**) liver tissue. Data are expressed as mean ± standard error (*n* = 8 in each group). Significance was established for fumonisin-intoxicated groups versus the control group (no FB) using a one-way ANOVA followed by a Dunnett’s post-hoc test (normally distributed data) or a Kruskal–Wallis ANOVA with a Dunn’s post-hoc test (for pairwise comparisons with at least one non-normally distributed dataset); * *p* < 0.05; ** *p* < 0.01; *** *p* < 0.001.

Basal histomorphometrical analysis of the intestinal mucosa of the duodenum showed that FB intoxication significantly reduced villus height/length in hens in the 1.0 mg/kg b.w. and 10.9 mg/kg b.w. FB groups compared to the control group. The thickness/width of duodenal villi was significantly reduced in all FB-intoxicated groups, irrespective of FB dose. Both the thickness/width and the depth of the duodenal crypts were also significantly reduced in all FB-intoxicated groups, irrespective of dose. There were no significant differences in the villus/crypt ratio between groups (Figure 3A).

The jejunal villus height/length was significantly reduced following FB intoxication, irrespective of FB dose, whereas the villus width/thickness was reduced following FB intoxication at a dose of 10.9 mg/kg b.w. compared to the control group. Both jejunal crypt depth and villus/crypt ratio were reduced in all FB-intoxicated groups, irrespective of dose, whereas crypt width was reduced compared to that in control hens only in the 10.9 mg/kg b.w. FB group (Figure 3B). Structural information obtained from the analysis of collagen fibers in PicroSirius Red stained sections of both parts of the intestine and the liver showed that both fiber types were present in the sections, with the predominance of mature, thick collagen fibers in all of the investigated sections in both the control and the FB-intoxicated groups of hens. A significant decrease in the percent of immature, thin collagen fibers was observed in the duodenum of hens in the 1.0 mg/kg b.w. FB group, whereas a significant increase in the percent of immature, thin collagen fibers was observed in the 10.9 mg/kg b.w. FB group compared to that observed in the control hens (Figure 3C). No changes in the immature collagen content of the jejunum were observed between groups (Figure 3D). A significant increase in the percent of immature collagen fibers was observed in the liver of hens in the 10.9 mg/kg b.w. FB group compared to the control group (Figure 3E).

**Figure 4:**
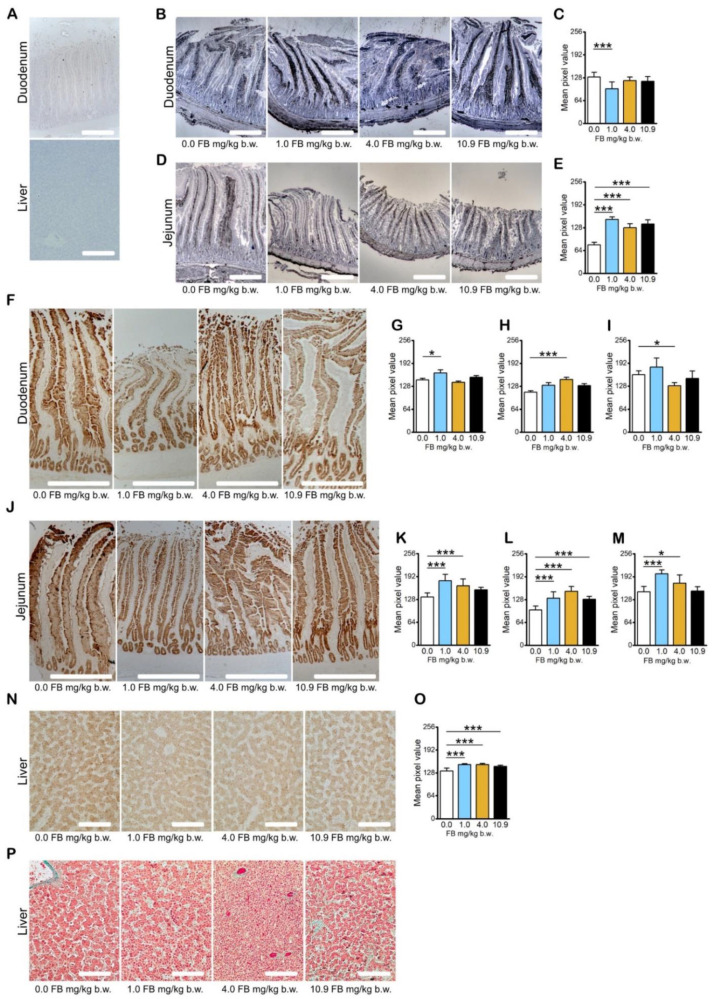
The effect of fumonisin intoxication on the expression of zonula occludens tight junction protein-1 (ZO-1) and claudin-3 (C-3) proteins in intestine and liver tissue in pre-laying hens. (**A**) Representative pictures of the antibodies’ control for immunohistochemical reactions in the intestine (duodenum) and liver; (**B**) representative photomicrographs of the immunohistochemical reactions for ZO-1 in the duodenum; (**C**) the intensity of ZO-1 expression in the duodenum, measured by comparison of the pixel brightness value in the microscopic images converted to 8-bit, grey-scale images (the higher the pixel value, the lower the intensity of the immunoreaction); (**D**) representative photomicrographs of the immunohistochemical reactions for ZO-1 in the jejunum; (**E**) the intensity of ZO-1 expression in the jejunum, measured by comparison of the pixel brightness value in the microscopic images converted to 8-bit, grey-scale images (the higher the pixel value, the lower the intensity of the immunoreaction); (**F**) representative photomicrographs of the immunohistochemical reactions for C-3 in the duodenum; the intensity of C-3 expression in (**G**) the whole duodenum, (**H**) the duodenal crypts, and (**I**) the duodenal villi measured by comparison of the pixel brightness value in the microscopic images converted to 8-bit, grey-scale images (the higher the pixel value, the lower the intensity of the immunoreaction); (**J**) representative photomicrographs of the immunohistochemical reactions for C-3 in the jejunum; the intensity of C-3 expression in (**K**) the whole jejunum, (**L**) jejunal crypts, and (**M**) jejunal villi, measured by comparison of the pixel brightness value in the microscopic images converted to 8-bit, grey-scale images (the higher the pixel value, the lower the intensity of the immunoreaction); (**N**) representative photomicrographs of the immunohistochemical reactions for C-3 in the liver; (**O**) the intensity of C-3 expression in the liver, measured by comparison of the pixel brightness value in the microscopic images converted to 8-bit, grey-scale images (the higher the pixel value, the lower the intensity of the immunoreaction); (**P**) representative photomicrographs of Goldner’s staining of the liver. Scale bars: A, B, D, F, J, N and P: 200 µm; A, F, J, N: sections developed in 3,3′-diaminobenzidine tetrahydrochloride (DAB). Counterstaining performed with Mayer’s hematoxylin; B, D: sections developed in 3,3′-diaminobenzidine tetrahydrochloride (DAB) with metal enhancer; counterstaining performed with nuclear fast red (NFR). C, E, G, H, I, K, L, M, O: data expressed as mean ± standard error (*n* = 8 in each group). Significance was established for fumonisin-intoxicated groups versus the control group (no FB) using a one-way ANOVA followed by a Dunnett’s post-hoc test (normally distributed data) or a Kruskal–Wallis ANOVA with a Dunn’s post-hoc test (for pairwise comparisons with at least one non-normally distributed dataset); * *p* < 0.05; *** *p* < 0.001.

The expression of zonula occludens tight junction protein-1 (ZO-1), a protein involved in signal transduction at cell–cell junctions, was significantly increased in the duodenum of hens in the 1.0 mg/kg b.w. FB group (Figure 4B,C; the higher the pixel value, the lower the intensity of immunoreaction) and significantly decreased in the mucosal epithelium of the jejunum of all FB-intoxicated hens compared to the control hens (Figure 3D,E).

The expression of claudin-3, the tight junction protein commonly expressed in the epithelia of the intestine and in the liver, was significantly decreased in the duodenal epithelium of hens in the 1.0 mg/kg b.w. FB group (Figure 4F,G). Claudin-3 expression was decreased in the duodenal crypts (Figure 4H) and increased in the duodenal villi of hens in the 4.0 mg/kg b.w. FB group compared to the control hens (Figure 3I). The expression of claudin-3 in the jejunal epithelium was significantly decreased in hens in the 1.0 mg/kg b.w. and 4.0 mg/kg b.w. FB groups (Figure 4J,K) compared to that observed in the control group. Claudin-3 expression was decreased in the duodenal crypts of hens in all FB-intoxicated groups (Figure 4L) as well as in the jejunal villi of hens in the 1.0 mg/kg b.w. and 4.0 mg/kg b.w. FB groups (Figure 4M) compared to the control hens. The expression of claudin-3 in the liver was significantly reduced in all FB-intoxicated groups (Figure 4N,O) compared to the control hens.

The epithelial integrity of the duodenum and jejunum was disrupted in all hens intoxicated with FB compared to the control hens, irrespective of the dose. Although the overall morphology of the various layers of the intestinal wall was preserved, and the general shape of the villi and crypts was also preserved, disruption of the villi epithelium in the apical and middle parts and intercellular gaps were clearly visible in the histological preparations.

Histological examination of the liver (Figure 4P) showed various degenerative changes dependent on the FB dose. Hepatocytes of hens in the 10.9 mg/kg b.w. FB group showed cytoplasmic vacuolization and signs of cirrhosis could be distinguished, whereas acidophilic bodies were present in the livers of hens in the 1.0 mg/kg b.w. FB group, signifying apoptotic hepatocytes grouped in abundance in small areas reflecting acute hepatocellular injuries. Hens in the 4.0 mg/kg b.w. FB group displayed swollen hepatocytes with ballooning degeneration.

### 2.3. Results of Bone Analyses

**Figure 5:**
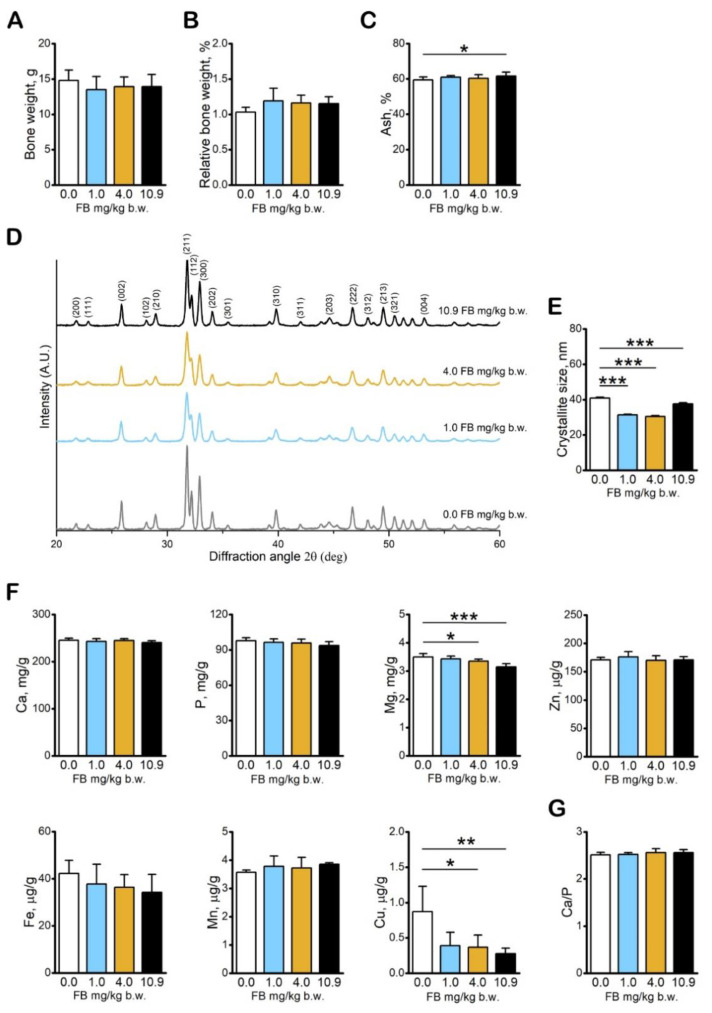
The effect of fumonisin intoxication on the bone weight and characteristics of the bone mineral phase in pre-laying hens. (**A**) Tibia weight; (**B**) relative tibia weight; (**C**) tibia ash percentage; (**D**) representative XRD diffractograms of bone samples, crystalline peaks corresponding to hydroxyapatite are indicated, and peaks corresponding to the (002) plane were used for calculations of crystallite size; (**E**) bone hydroxyapatite crystallites size; (**F**) macro- and microelement content in tibia; (**G**) bone Ca/P ratio. A, B, C, E, F, G: data are expressed as mean ± standard error (*n* = 8 in each group). Significance was established for fumonisin-intoxicated groups versus the control group (no FB) using a one-way ANOVA followed by a Dunnett’s post-hoc test (normally distributed data) or a Kruskal–Wallis ANOVA with a Dunn’s post-hoc test (for pairwise comparisons with at least one non-normally distributed dataset); * *p* < 0.05; *** *p* < 0.001.

FB intoxication did not influence actual bone weight (Figure 5A) or relative bone weight (Figure 5B) of the growing hens. Bone ash content was significantly decreased in hens in the 10.9 mg/kg b.w. FB group (Figure 5C) compared to the control hens. X-ray diffraction analysis (Figure 4E) revealed that fumonisin intoxication affected the structural organization of the bone mineral phase, significantly lowering the size of hydroxyapatite crystallites in all FB-intoxicated hens (Figure 5D,E). Among the analyzed bone macro and microelements, a significant decrease in Cu and Mg was noted in hens following intoxication with FB at doses of 4.0 mg/kg b.w. and 10.9 mg/kg b.w.; no other changes in bone macro- and microelements were observed (Figure 5F). Fumonisin intoxication had no significant effects on the bone Ca/P ratio (Figure 5G).

**Figure 6:**
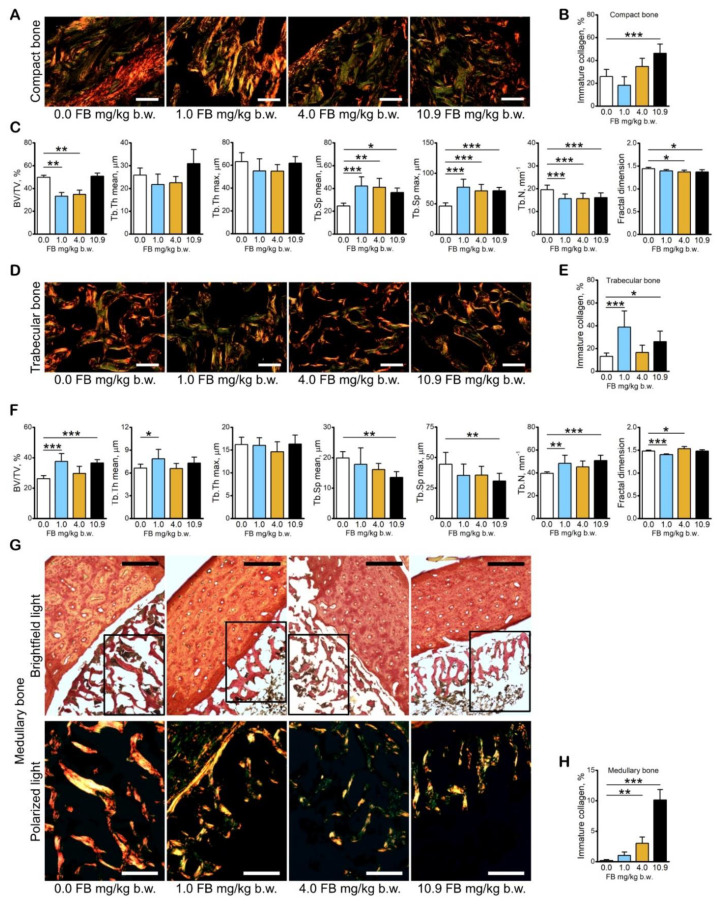
The effect of fumonisin intoxication on the bone content of immature collagen and bone histomorphometry in pre-laying hens. (**A**) Representative pictures of the distribution of thin, immature collagen fibers in PRS-stained sections of compact bone observed in polarized light; (**B**) content of thin, immature collagen fibers in compact bone; (**C**) histomorphometric analysis of trabecular bone; (**D**) representative pictures of the distribution of thin, immature collagen fibers in PRS-stained sections of trabecular bone observed in polarized light; (**E**) content of thin, immature collagen fibers in trabecular bone; (**F**) histomorphometric analysis of medullary bone; (**G**) representative pictures of the distribution of thin, immature collagen fibers in PRS-stained sections of medullary bone observed in brightfield light (the marked sections are presented in bottom panels in polarized light); (**H**) content of thin, immature collagen fibers in medullary bone. Scale bars: A and C: 20 µm; G: 200 µm upper panels and 100 µm bottom panels. B, C, E, F, H: data are expressed as mean ± standard error (*n* = 8 in each group). Significance was established for fumonisin-intoxicated groups versus the control group (no FB) using a one-way ANOVA followed by a Dunnett’s post-hoc test (normally distributed data) or a Kruskal–Wallis ANOVA with a Dunn’s post-hoc test (for pairwise comparisons with at least one non-normally distributed dataset); * *p* < 0.05; ** *p* < 0.01; *** *p* < 0.001.

Structural information pertaining to the bone organic phase, obtained from the analysis of collagen fibers in PRS-stained sections of bone tissue, showed that both mature and immature collagen fibers were present, with a predominance of the mature, thick (red) collagen in compact (Figure 6A), trabecular (Figure 6D), and medullary bone (Figure 6G) of control and FB-intoxicated hens. However, an increase in immature, thin (green) collagen fibers was observed in the compact bone of hens in the 10.9 mg/kg b.w. FB group (Figure 6B), in the trabecular bone of hens in the 1.0 mg/kg b.w. and 10.9 mg/kg b.w. FB groups (Figure 6E), and in the medullary bone of hens in the 4.0 mg/kg b.w. and 10.9 mg/kg b.w. FB groups (Figure 6H) compared to the control hens.

Histomorphometrical analysis of trabecular bone (Figure 6C) showed a significant decrease in bone volume (BV/TV) in hens in the 1.0 mg/kg b.w. and 4.0 mg/kg b.w. FB groups caused by an increase in the mean and maximal trabecular space as well as a decrease in trabecular number compared to that observed in the control group. The mean and maximal trabecular space increased, whereas the trabecular number decreased in hens in the 10.9 mg/kg b.w. FB group (Figure 6C) compared to the control hens. The fractal dimension decreased significantly following intoxication with FB at doses of 4.0 mg/kg b.w. and 10.9 mg/k b.w. compared to the control hens. No significant changes in mean and maximal trabecular thickness in the trabecular bone of FB-intoxicated hens were observed.

Histomorphometrical analysis of medullary bone indicated a significant increase in bone volume in hens in the 1.0 mg/kg b.w. FB group as a consequence of the increased number and mean thickness of the medullary trabeculae (Figure 6F). Moreover, a significant increase in medullary bone volume, with increased number, mean, and maximal space, was observed in hens in the 10.9 mg/kg b.w. FB group compared to the control hens. The fractal dimension was significantly decreased after intoxication with FB at a dose of 1.0 mg/kg b.w. and was increased in hens following intoxication with 4.0 mg/kg b.w. FB compared to the control group. No other changes in the medullary bone were observed in FB-intoxicated hens.

**Figure 7:**
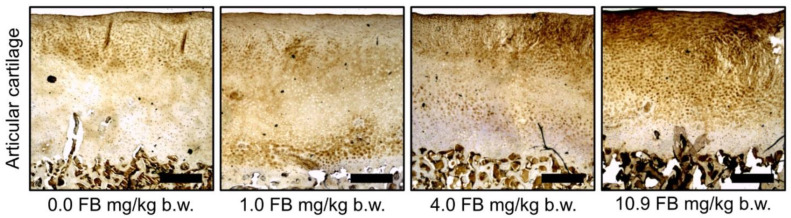
The effect of fumonisin intoxication on the distribution of cartilage oligomeric matrix protein (COMP) in tibial articular cartilage of pre-laying hens. Representative pictures of the immunohistochemical analysis of COMP carried out on formaldehyde-fixed sections from the tibial articular cartilage of laying hens from the control (no FB) and fumonisin-intoxicated groups. Cartilage from hens in the 10.9 mg/kg b.w. FB group showed a denser and darker staining, indicating higher COMP deposition, especially in the upper zones of the cartilage. All scale bars represent 10 μm.

The analysis of cartilage oligomeric matrix protein (COMP) immunostaining in articular cartilage showed differences in the quantity and distribution of COMP between the different dietary groups (Figure 7). Cartilage from hens in the control group, as well as cartilage collected from hens intoxicated with FB at doses of 1.0 mg/kg b.w. and 4.0 mg/kg b.w., showed a lower accumulation of COMP (brown color) through the whole thickness of the cartilage, whereas cartilage from the hens intoxicated with FB at a dose of 10.9 mg/kg b.w. showed a denser and darker brown staining, indicating higher COMP deposition, especially in the upper zones of the cartilage.

## 3. Discussion

The presence of FBs in cereal grains (including maize), crop products, and pelleted feed is common. Thus, in order to prevent the involuntary intake of FBs and the resultant adverse effects, numerous countries regulate FB concentrations in feed and agricultural commodities [5,12,13]. On the other hand, many countries also have no set maximum values for consumption, not only for FBs but also for mycotoxins in general, and they establish their own regulation limits in both human foods and animal feed.

Previous studies concerning the gut–bone axis in laying hens are limited, and there are no studies, to our knowledge, in growing hens during the pre-laying period. The intestinal tract is the primary site at which the body comes into contact with dietary antigens and toxins, which are not only capable of triggering an inflammatory response but also of disrupting the digestive process, changing the structure of the intestines, impairing absorption, and, consequently, affecting bone homeostasis [14,15]. The adverse effects following the oral administration of FB in poultry are dependent on the species. The oral bioavailability of FB in laying hens, administered with FB at a dose of 2 mg/kg b.w., is similar to that noted in turkeys and ducks after FB administration at a dose of 100 mg/kg b.w. [8]. Kubena et al. [16] observed no mortality in mature laying hens following several weeks of FB intake at a dose of 200 mg/kg feed, which corresponds to about 10 mg/kg b.w. However, in the same study, there was a 20% mortality rate in adult laying hens (24-week-olds at the start of FB administration) following FB intoxication at a dose of 100 mg/kg feed [16]. Ledoux et al. (1992) demonstrated that FB doses of up to 300 mg/kg feed are needed to trigger clinical toxicity and decreased weight gain in broiler chickens [17]. A decrease in body weight was observed in one-day-old chickens intoxicated with FB at a dose of 600 mg/kg feed [18]. The resulting decrease in body weight following FB administration in various avian species is well described in the literature and said to be a consequence of decreased feed intake and poor feed efficiency [19,20,21,22]. Our results are consistent with these reports. In the current study, FB intoxication was performed by the oral administration of FB, not through the consumption of FB-contaminated feed. Nevertheless, a decrease in final body weight was observed in the young, growing hens intoxicated with FB compared to the control hens, irrespective of FB dose. Previous studies have yielded opposing results, where no changes in body weight were observed following FB intoxication [23,24]. The variability in results with regards to changes in body weight observed following FB intoxication is probably due to differences in the species and the age of the birds used in the studies, both of which could influence the development of the deleterious effects following FB intoxication. In the current study, growing hens (11 weeks of age at the start of the experimental period) were used during the pre-laying period. We observed changes in the number of erythrocytes and leukocytes in FB-intoxicated hens, which were dependent on the FB dose. Only one previous study assessed basal blood morphological parameters in 21-day-old broilers fed naturally contaminated feed (5.87 mg/kg feed), and their results were consistent with some of our results [25]. The authors reported an increase in hematocrit and hemoglobin concentration following FB intoxication; however, these parameters were unchanged in our study. They also noted a decrease in mean erythrocyte volume following FB intoxication [25]. A few studies performed in chickens and/or other poultry species have observed an increase in serum ALT, AST, ALP, and creatine kinase or a decrease in total protein, irrespective of the manner of FB intoxication, age of the birds, dose of FB intoxication, or the duration of the experimental period (period of FB intoxication) [13,15,20,25,26,27]. Another study involving 37-week-old laying hens intoxicated with FB at a dose of 25 mg/kg feed showed no effects of FB intoxication on the plasma concentration of total cholesterol, which is similar to our results [28]; although, our study was performed on pre-laying, 11-week-old hens. Chowdhury and Smith [29] observed no change in glucose concentrations, increased serum uric acid, and decreased serum cholesterol and Ca concentrations in 45-week-old laying hens fed feed naturally contaminated with FB for a period of 4 weeks [29]. Dazuk et al. [22] observed no significant changes in serum total protein and ALT concentrations in 25-week-old laying hens following 28 days of FB intoxication. However, in contrast to our study, they observed a decrease in serum ALP activity and an increase in serum ALT activity following 84 days of feeding 25-week-old laying hens a diet contaminated with 4 ppm T-2 and 20 ppm FB1 [22]. The level of serum alanine aminotransferase (ALT) activity reflects damage to hepatocytes and is considered to be a highly sensitive and fairly specific preclinical and clinical biomarker of hepatotoxicity. Damaged hepatocytes release their contents, including ALT and AST, into the extracellular space. However, serum AST activity is considered a less specific biomarker of liver function compared to ALT activity. Alkaline phosphatase level is also considered to be a marker of hepatocyte damage and is the primary marker of adverse hepatobiliary effects [30]. However, with hepatocyte damage, increases in serum ALT or LDH are not always observed. Henry et al. [23] observed no changes in serum ALP, LDH, total protein, glucose, cholesterol, uric acid, Ca, and K in broiler chickens following 21 days of FB intoxication at a dose of 80 mg/kg feed. In the current study, the serum concentrations of the mineral assessed indicated that, in general, mineral homeostasis was maintained, despite a surprising decrease in serum Ca in hens intoxicated with FB at a dose of 1.0 mg/kg b.w., which was not observed in hens intoxicated with FB at the highest dose. Additionally, bone Ca and P content, the Ca/P ratio, and bone weight were unaffected by FB intoxication in our hens, while a decrease in bone ash Cu and Mg content was noted following FB intoxication, as revealed by ICP-OES analysis. The opposite effect was observed in 34-day-old broilers intoxicated with FB at a dose of 10 mg/kg feed, where tibia ash Mg content was not affected by FB administration [31].

Both Cu and Mg play an essential role in cellular metabolism as well as in bone development [32,33,34]. Abnormal skeletal development continues to be a major problem and one of the primary causes of mortality in all commercial types of hens, which suffer excessive bone loss and fracture, which are mainly detected only after slaughter. Bone weakness and fragility in laying hens can result from decreased bone mass during the pre-laying period and can be intensified as a pathological condition throughout laying, causing economic losses and negatively affecting the welfare of the birds. Mycotoxin intoxication is linked with a reduction in feed intake and growth rate resulting in disturbances in bone homeostasis [35,36,37].

The findings of the current study were somewhat unexpected. FB intoxication was found to influence trabecular bone only negatively when administered at lower doses. It was surprising that FB administered at a dose of 10.9 mg/kg b.w. did not affect trabecular bone volume. An increase in trabecular thickness, albeit not statistically significant, was sufficient to maintain the same BV/TV, even when trabecular space increased. Fractal dimension measurements reflect the roughness of the bone image texture, which is related to the bone microarchitecture and is an indicator of intensity of bone loss [38,39,40]. Lower fractal trabeculae dimensions of FB-intoxicated pre-laying hens related to the complex bone structure indicated a greater loss of bone. The current study also revealed an increased amount of immature collagen in trabecular and compact bone, which could indicate intense bone repair or bone turnover. The same result was observed in the medullary bone of the pre-laying hens, which is a secondary bone in the marrow cavity and plays a key role in the maintenance of bone homeostasis in layers during eggshell formation [36,41]. Besides an increase in the content of immature collagen in medullary trabeculae, we also observed an improvement in all the histomorphometrical parameters assessed. Collagen provides support to hydroxyapatite, and it is amongst the collagen that the hydroxyapatite crystals are deposited and calcification occurs. FB intoxication, irrespective of the dose, resulted in reduced hydroxyapatite crystallites size, which could lead to a changes in bone material properties [42,43] as too small crystallites do not reinforce the bone composite material [44]. This effect of FB intoxication on hydroxyapatite crystallites size was previously reported in rats, where a reduction of bone mechanical properties was observed [7].

As previously discussed, an increased amount of immature collagen fibers was observed in the bone tissue of our pre-laying hens intoxicated with FB, which is indicative of intensified bone turnover. A similar effect was observed in the articular cartilage of hens intoxicated with 10.9 mg/kg b.w. FB, where COMP expression was increased, indicating enhanced fibrillogenesis.

It was shown that changes in GIT function are linked with changes in bone homeostasis and bone properties [14,45]. Changes in the structure of the intestinal mucosa results in changes in digestive and absorptive processes within the intestine, which in turn influence general metabolism, including that of bone tissue. Additionally, when toxic factors, e.g., FBs, are present in animal feed, disturbances in both the liver and kidney tissues are observed, over and above the structural changes in the intestinal mucosa. There are numerous previous studies that have confirmed the detrimental effects of FB intoxication on the kidneys, liver, and intestinal mucosa of poultry. FB intoxication, even at a relatively low dose, e.g., 100 mg/kg feed, has been shown to cause diarrhea in chickens and turkeys [17,27]. Changes in villi or crypt morphology are representative of the enteroplasticity of the intestine, a manifestation of the ability of the intestines to adapt to various conditions [16,31,46,47,48,49,50]. The results of the current study, regarding the structural changes observed in the intestinal mucosa of the pre-laying hens, are consistent with that of previous studies. However, in the current study, we also demonstrated changes in the expression of integral proteins within the intestinal barrier, which is formed by a complex multi-protein network between the epithelial cells including tight junctions, adhering junctions, and gap junctions, which maintain the mechanical integrity of the intestine and are responsible for various properties and functions of the intestinal epithelium. In the current study, integral transmembrane, tight junction proteins, like zonula occludens were assessed. These tight junction proteins serve as a barrier to adverse luminal agents and allow for the permeation of ions, solutes, and water [50]. Claudin-3, another tight junction protein that was assessed in the current study, plays a key role in the maintenance of vital functions and homeostasis within the cell. The modification of tight junction barrier function is dynamically regulated by various extracellular factors, including mycotoxins, and is strictly associated with health and general homeostasis [7,50,51]. Our study showed that FB intoxication degraded ZO-1 in the jejunal epithelium, irrespective of the dose, whilst the opposite effect was observed in the duodenum, where only one dose of FB (1.0 mg/kg b.w.) effectively influenced ZO-1 expression, leading to the strengthening of the barrier by this protein. This effect has never been observed previously and should be further investigated. The expression of claudin-3 was also generally degraded in the jejunal epithelium of our pre-laying hens. Interestingly, significantly increased expression of claudin-3 was observed in the villi of our pre-laying hens that were intoxicated with FB at a dose of 4.0 mg/kg bw. Sometimes, chronic exposure to toxins at a relatively low dose can cause more detrimental effects than that following intoxication with higher doses. This phenomenon should be further investigated.

FB toxicity in quantities that did not cause any evident acute toxicity for 21 days in the current study (FB quantities were in line with the proposed value for fumonisins B1 and B2 in products intended for animal feed in the EU) was enough to trigger significant negative changes in the intestinal epithelium of the pre-laying hens, increasing their risk of the development of diseases such as cancer later in life [52].

## 4. Conclusions

In conclusion, this is the first report showing the effects of FB intoxication on enteroplasticity, liver, and bone tissue in pre-laying hens. Despite the improvement in some of the histomorphometrical parameters assessed in medullary bone, FB intoxication leads to negative structural changes in bone tissue in young hens, which can result in worsening bone mechanics and the increased risk of bone fracture. Moreover, our study showed that FBs, even at a very low dose, might degrade the intestinal barrier and predispose the animals to intestinal disturbances later in life.

## 5. Materials and Methods

All chemicals and solvents were of analytical reagent grade and were obtained from Sigma-Aldrich (St. Louis, MO, USA), Thermo Fisher Scientific (Waltham, MA, USA), or Avantor Performance Materials Poland S.A. (Gliwice, Poland).

### 5.1. Fumonisins

FB were biosynthesized in vitro in house on a maize grain medium with the use of *F. moniliforme* obtained from the biobank at the Laboratory of Mycotoxicology, Institute of Veterinary Medicine of The National Academy of Agrarian Sciences of Ukraine, Kiev, Ukraine. Cultures were inoculated and cultivated for 4 weeks on autoclaved, coarsely cracked, maize grains in the dark at a temperature of 24 °C. Incubation was then terminated and the contaminated maize was collected and autoclaved at 121 °C for 15 min. The samples were thereafter dried between 80–90 °C for 120 min, ground, and stored at −20 °C. Samples were analyzed for FB1 and FB2 by liquid chromatography conducted at the National Veterinary Research Institute, Puławy, Poland (AOAC International, method #2001.04). FB were detected at 2.07:1.00 ratios for FB1 and FB2, respectively (73% and 27%, respectively). No detectable amounts of fusariotoxins (zearalenone, deoxynivalenol, and fusarochromanone) were noted. For dietary treatment, contaminated ground maize grains were extracted with an ethanol:water solution (70:30 v:v). The obtained extract was then filtered and analyzed for FB concentration using an ELISA (Ridascreen Fumonisin, #R3401, detection limit: 25 ug/kg; R-Biopharm AG, Darmstadt, Germany), according to the manufacturer’s protocol. The extract was then concentrated to 100 mg/mL by evaporation in a rotary evaporator. During the experiment period, the obtained FB1+FB2 extract stock was diluted in distilled water to yield the necessary concentration in 1 mL, on the basis of individual hen weight, which was recorded daily.

### 5.2. Animals, Experimental Procedures, and Tissue Collection

The study was conducted under vivarium conditions at the State Scientific-Research Control Institute of Veterinary Medicinal Products and Feed Additives in Lviv, Ukraine. The experiment was carried out using 32, 9-week-old Isa Brown hens obtained from a commercial source. Hens were housed in a poultry house in individual cages (50 × 62 × 41 cm) on a wire-mesh floor under controlled climate conditions (temperature 19 °C +/− 2 °C; 50–65% relative humidity; 10 lux light intensity). Housing and feeding conditions were all in accordance with the Council 105 Directive 1999/74/EC of the 19th of July of 1999, which states the minimum standards for the protection of laying hens. The hens were allowed a 2-week acclimatization period, during which water and control feed were given ad libitum.

During the acclimatization period, which lasted from the 9th to the 11th week of age, all hens were fed the same pre-laying, standard, commercial diet (supplementary Table S1), which was the same as that used during the experimental period. The composition and nutrient levels of all diets met the nutrient specifications according to the Polish-recommended requirements [53]. The experimental period took place during the growing period of the hens, from the 11th to the 14th week of age. During the experimental period the hens had free access to standard commercial laying hens’ mash feed and water and were exposed to a 14 L:10 D lighting schedule. At the start of the experimental period, hens were randomly assigned to one of four experimental groups and individually weighed. The weight of hens in each of the experimental groups, each of which compromised 8 hens, was not significantly different (943 ± 91 g; 951 ± 39 g; 925 ± 68 g; 937 ± 27 g). The hens were orally administered (to the crop) 1.0 mg/kg b.w., 4.0 mg/kg b.w., or 10.9 mg/kg b.w. of the FB1+FB2 extract for up to 21 days. The FB1+FB2 extract was dissolved in distilled water. Hens in the control group (which did not receive the extract) were treated with an equal amount of distilled water (1 mL). Water and feed were provided ad libitum. The basal diet was considered free from mycotoxins, as liquid chromatography analysis showed that it contained 53 µg/kg of FB1+FB2, which was below the maximum levels allowed by the EU regulations.

The 1.0 and 4.0 mg/kg b.w. doses of the FB1+FB2 extract were chosen based on the literature, where the selected criterion was the NOAEL and LOAEL in chickens [5,8,54]. The 1.0 mg/kg b.w. dose corresponded to a dose of 20 mg/kg feed, which is identified as a NOAEL for chickens and is in line with the guide value for fumonisins B1 and B2 in products intended for animal feed in the EU [4]. The 4.0 mg/kg b.w. dose was lower than a LOAEL determined for chickens (4.7 mg/kg b.w./per day corresponds to 40 mg/kg feed) [5]. In accordance with the feed intake in our pre-laying hens, the 4.0 mg/kg b.w. dose corresponded to a dose of 80 mg/kg feed, which has been shown to result in disturbed liver function in chickens [5]. The 10.9 mg/kg b.w. dose was established in a preliminary study, which lasted for 21 days, where the median lethal dose for oral FB exposure (LD_50_ = 218 mg/kg b.w.) was determined and was found to be equal to 0.2 of the established LD_50_ value. In accordance with the feed intake of our hens, the 10.9 mg/kg b.w. dose corresponded to a dose of 218 mg/kg feed.

At the end of the experimental period, all hens were weighed and slaughtered via cervical dislocation and decapitation after electrical stunning. Blood was collected post-mortem from all slaughtered birds, and the serum was separated by centrifugation at 3000 rpm for 15 min at 4 °C and stored at −20 °C until further analysis. Liver, duodenum, and jejunum samples were then collected and immediately prepared for histological investigation as described below. Finally, both the tibiae were carefully dissected out and cleaned of adhering tissues.

### 5.3. Basal Blood Morphology and Blood Serum Analyses

Blood hematological parameters (total white blood cells, hemoglobin, hematocrit) were assessed using a hematology analyzer (Mindray BC-2800Vet, Bio-Medical Electronics, Shenzhen, China); red blood cells were manually counted. Blood serum concentrations of total protein, glucose, uric acid, total cholesterol, K, Mg, Ca, and P and the activities of alkaline phosphatase (ALP), aspartate aminotransferase (AST), alanine aminotransferase (ALT), lactate dehydrogenase (LDH), and creatine kinase were determined using an automated biochemical analyzer (HumaLyzer 3000; Human GmbH, Weisbaden, Germany) and sets of ready-to-use, commercially available biochemical reagent kits (Human GmbH, Weisbaden, Germany), according to the manufacturer’s protocols.

### 5.4. Analyses of Intestine and Liver Samples

Duodenal (30 mm long; obtained 20 mm distal to the pylorus) and jejunal segments (30 mm long; 50% of the total intestinal length of the jejunum), as well as 1 cm^3^ samples from the right lobe of the liver, were obtained from each bird immediately after slaughter. The tissues were rinsed with physiological saline and fixed in phosphate-buffered 4% paraformaldehyde (pH 7.0) for 48 h. The samples were then dehydrated through a graded ethanol series (50%, 70%, 90%, and 100% ethanol in distilled water), fixed with nonpolar Ottix Plus and Ottix Sharper solvents (DiaPath, Martinengo, Italy), and embedded in paraffin. Cross sections of 5 μm thick were cut with a HM 325 microtome (Thermo Fisher Scientific, Waltham, MA, USA) and stained with Goldner’s trichrome (liver) and PicroSirius Red (PSR) (liver and intestine).

Immunohistochemical staining (zonula occludens 1, ZO-1: intestine; claudin-3, C-3: intestine and liver) was performed after deparaffinization in xylene and rehydration with decreased concentrations of ethanol and distilled water. Heat-induced epitope retrieval was performed in sodium citrate buffer (10 mM sodium citrate, 0.05% Tween 20, pH 6.0). using a pressure cooker, Rapid Cook (Morphy Richards, Swinton, UK). Endogenous peroxidase activity was subsequently blocked with a 3% solution of hydrogen peroxide in deionized water for 5 min. After blocking for 30 min in normal serum, sections were incubated with the primary antibodies over night at 4 °C. All primary antibodies were chicken-specific: rabbit polyclonal anti-C-3 antibody (ab15102; Abcam, Cambridge, UK, dilution 1:100) and rabbit polyclonal anti-ZO-1 (antibody (orb311587, Biorbyt, St. Louis, MO, USA, dilution 1:500) were used. The sections were then incubated for 30 min with the appropriate secondary antibodies. Negative control sections for each antibody were obtained by identical immunohistochemical staining, excluding the primary antibody (Figure 4A). The C-3 sections were then developed in 3,3′-diaminobenzidine tetrahydrochloride (DAB D5905; Sigma-Aldrich, St. Louis, MO, USA); ZO-1 sections were developed using 3,3′-diaminobenzidine tetrahydrochloride with a metal enhancer (SIGMAFAST™ DAB D0426; Sigma-Aldrich, St. Louis, MO, USA), both used as chromogens for 15 min at room temperature. Counterstaining was performed with Mayer’s hematoxylin (MHS32-1L; Sigma-Aldrich, St. Louis, MO, USA) or Nuclear Fast Red counterstain (H-24-2; Vector Laboratories Inc., Burlingame, CA, USA) [55,56].

Microscopic slides were observed in brightfield (Goldner’s trichrome, immunohistochemistry) and cross-polarized light (PRS) using a CX43 (Olympus, Tokyo, Japan) microscope at the following magnifications: x2.5, x20, and x40. The microscopic images obtained were then examined using graphical analysis software: Olympus cellSens (Olympus, Tokyo, Japan) and ImageJ [57].

The following morphometric parameters were analyzed in the intestinal sections obtained: villus thickness and length, crypt width and depth, and the villus length to crypt depth ratio. The measurements of each variable were made on three separate tissue sections, on at least ten different areas of each section. The measurements were then averaged and expressed as the mean value of each parameter for each hen.

Microscopic observations of Goldner’s-trichrome-stained liver samples allowed us to identify and assess the structure and morphology of the liver; observation of PSR-stained sections of the intestine and liver was used to differentiate collagen fibers, where mature, organized, thicker collagen fibers are seen as red/orange and immature, thinner fibers are seen as green in polarized light [58,59]. A color threshold tool In ImageJ was used to calculate the area of immature and mature collagen fibers in selected image sections using a pixel counting method.

The intensity of the immunoreaction (grey or brown color, depending on the staining) was measured by the quantitative comparison of the mean pixel intensity value in the photomicrographs converted using ImageJ into 8-bit, grey-scale images, with the scale from 0 (white pixel) to 255 (black pixel); the lower the pixel value, the higher the intensity of immunohistochemical reaction [56,60]. The intensity of the immunoreaction in each of the analyzed digital images was measured in 10 randomly selected areas of the positive signal. All the analyses were done blindly by an associate who was not aware of the treatment.

### 5.5. Analyses of Bone Samples

A 30-mm long, transverse section from the right tibia diaphysis was cut using an diamond bandsaw (MBS 240/E, Proxxon GmbH, Foehren, Germany). The samples were cleaned of the bone marrow under running water and then defatted using chloroform-methanol (2:1, v:v) at room temperature for 24 h under constant agitation using an Elpan 357 laboratory shaker (Elpin-plus, Lubawa, Poland). After drying for 24 h at a temperature of 105 °C, they were calcined in a muffle furnace at 500 °C for 24 h. The bone ash percentage was expressed relative to the dry mass of the bone. Ashed bone samples were placed in a mortar and pulverized, using a pestle, into a fine white powder. The powdered bone ash was transferred to two 2.0 mL Eppendorf microtubes. Bone samples from the first microtube were used for XRD measurements and those from the second microtube for ICP-OES spectrometry.

The mean size of the hydroxyapatite of the bone ash was measured using an X-ray diffraction (XRD) method performed with a high-resolution Empyrean powder diffractometer (PANalytical, Almelo, The Netherlands) with Cu K-alpha radiation (λ = 1.54178 Å). Samples were measured in θ–2θ geometry, over a range from 10 to 80°, with a step size of 0.01° and a counting time of 6 s per data point. All measurements were carried out at room temperature. Bragg peaks and crystallographic planes were identified using a HighScore Plus software package (PANalytical, Almelo, Netherlands) from the hydroxyapatite references (No. 96-901-0053). The sizes of the hydroxyapatite crystallites on the c-axis were calculated using the Miller index (002). The peak position and the full-width at half-maximum (FWHM) intensity were calculated from the fits of the Gaussian function to each (002) peak using OriginPro 2016 software (OriginLab Co., Northampton, MA, USA). The mean size of the crystallites was calculated according to the Scherrer equation [61] with the shape constant of 0.9 and an apparatus broadening of 0.01°.

The mineral composition of the ashed bone samples was determined using inductively coupled plasma—optical emission spectrometry (ICP-OES, iCAP Series 6500, Thermo Scientific, Waltham, MA, USA). The TraceCERT multi-element stock solution (Sigma-Aldrich, St. Louis, MO, USA) was used to prepare reference standards. The content of macro- and microelements was expressed as in defatted, dried bone [62].

Bone sections for histomorphometry were obtained from the left tibia diaphysis and proximal metaphysis. Samples of the lateral condyle (5-mm thick) were cut perpendicularly to the articular surface using an MBS 240/E diamond bandsaw. Similarly, 5-mm thick samples of the bone diaphysis, containing medullary bone, were cut at the midpoint of the bone diaphysis. Bone samples were fixed in formaldehyde (4%, pH 7.0) and then decalcified in EDTA (10%, pH 7.4) and dehydrated through a graded ethanol series (50%, 70%, 90%, and 100%). The samples were then fixed with nonpolar Ottix Plus and Ottix Sharper solvents, embedded in paraffin, and cut with a microtome at a thickness of 4 μm. Slides were stained with PSR stain in order to evaluate basal morphology and the distribution of immature collagen fibers. The stained slides were observed in brightfield (histomorphometry) and cross-polarized (collagen distribution) light using an Olympus CX43 microscope (Olympus, Tokyo, Japan). ImageJ software was used to assess the morphology of the trabecular and medullary bone (relative bone volume, BV/TV; mean and max trabecular thickness, Tb.Th; mean and max trabecular space Tb.Sp; trabecular number, Tb.N) and to calculate the area of immature collagen fibers in selected image sections [60,63].

Immunohistochemical staining of articular cartilage sections was performed according to the protocols provided by the producer of the antibody. Slides were deparaffinized in xylene and rehydrated; endogenous peroxidase activity was then blocked with 3% H_2_O_2_ (Sigma-Aldrich, St. Louis, MO, USA). The enzymatic antigen retrieval step was performed by a 10-min incubation with proteinase K (Sigma-Aldrich, St. Louis, MO, USA) at 37 °C. The primary antibody targeting cartilage oligomeric matrix protein (Elabscience Biotechnology Co., Ltd., Wuhan, China, dilution 1:100 in diamond antibody diluent, Cell Marque Corp., Rocklin, CA, USA) was incubated at room temperature for 1 h. Ready-to-use Bright Vision +Poly-HRP-Anti Ms/Rb IgG Biotin-free (Immunologic, Duiven, The Netherlands) served as the secondary antibody. DAB (Abcam, Cambridge, UK) was used as a substrate-staining chromogen. Slides were counterstained with Mayer’s hematoxylin (MHS32-1L Sigma-Aldrich, St. Louis, MO, USA). Slides were examined using an Olympus CX43 microscope (Olympus, Tokyo, Japan) [40].

### 5.6. Statistical Analysis

Each individual bird was considered as an experimental unit. All statistical procedures were conducted using Statistica 13 software (TIBCO Software Inc., Palo Alto, CA, USA). A normal distribution of data was examined using the Shapiro–Wilk test, and equality of variance was tested by Levene’s test. For normally distributed data, a one-way analysis of variance (ANOVA) with Dunnett’s post-hoc test was used to compare FB groups with the control (no FB) group. For data that did not meet the assumptions for parametric tests, a non-parametric Kruskal–Wallis ANOVA with Dunn’s multiple comparisons test was used. For all tests, a p-value of less than 0.05 was considered statistically significant. The results were expressed as mean ± standard error (SE).

## Data Availability

The data presented in this study are available on request from the corresponding author.
